# Effects of Salts on Structural, Physicochemical and Rheological Properties of Low-Methoxyl Pectin/Sodium Caseinate Complex

**DOI:** 10.3390/foods10092009

**Published:** 2021-08-26

**Authors:** Shengyu Fan, Fang Fang, Ailing Lei, Jiong Zheng, Fusheng Zhang

**Affiliations:** 1 College of Food Science, Southwest University, Chongqing 400715, China; swufan@email.swu.edu.cn (S.F.); lal0422@email.swu.edu.cn (A.L.); zhengjiong_swu@126.com (J.Z.); 2 Whistler Center for Carbohydrate Research and Department of Food Science, Purdue University, West Lafayette, IN 47906, USA; ffang@purdue.edu

**Keywords:** low methoxyl pectin, sodium caseinate, complex, salt ion, stability

## Abstract

The addition of salts is an effective way to improve the properties of polysaccharide/protein complexes for use in foods. However, there is no comparative study on the effects of different ions on the complex system of low methoxyl pectin (LMP)/ sodium caseinate (CAS) complex. The effects of different concentrations of three salt ions (Na^+^, K^+^, Ca^2+^) on the physicochemical and rheological properties of the LMP/CAS complex were determined in this study, and the structure of LMP/CAS complex was characterized. The results showed that the addition of these three salt ions affected zeta potential, particle size, and turbidity of the LMP/CAS complex, and lead the LMP/CAS complex to form a more regular and uniform network structure, which helped improve its stability, solubility, and rheological properties. The particle size and turbidity value of the complex achieved with Ca^2+^ were higher than those obtained using Na^+^ and K^+^. Moreover, the secondary structure of the proteins in the complex changed to adding high concentrations of Ca^2+^. Our study provides valuable information for the application of the LMP/CAS complex in the food industry.

## 1. Introduction

The functional characteristics of food complexes composed of polysaccharides, proteins, lipids, and other biological macromolecules are significantly different from those composed of single components [1,2] and have attracted increasing attention in the food industry. In a polysaccharide/protein complex system, the polysaccharide can alter the physical and chemical properties and the functional characteristics of proteins and improve their stability near the isoelectric point, thereby enhancing the physical, chemical, and rheological properties, as well as the stability and texture of the complex [3]. Therefore, researchers have studied and developed a large number of polysaccharide/protein complexes and promote their use in the food industry, in the formulation of novel gels [4], emulsion stabilizers [5], complex edible films [6], and microcapsules [7].

Many studies have reported the effects of single ions on the properties of the polysaccharide/protein complex, which showed that the formation of a polysaccharide/protein complex involves non-covalent interactions, mainly electrostatic interactions [8,9]. Salt ions affect the properties of the polysaccharide/protein complex [10,11] and partially shield the charges on these macromolecules and affect their interactions [12]. Additionally, different salt ions have different effects during the complex formation [13].

Sodium caseinate (CAS) is widely used as an emulsifier, thickener, and stabilizer in the food industry [14], but its application is limited owing to its instability and coagulation under acidic conditions. Research showed that many hydrophilic polysaccharides, such as high methoxyl pectin [15], carboxymethyl cellulose [16], and sodium alginate [17] can improve their stability. Low methoxyl pectin (LMP) is a water-soluble polysaccharide with good gelling properties with the presence of some salt ions, such as Ca^2+^, over a wide pH range. Under acidic conditions, LMP and CAS carries opposite charges. Thus, negatively charged pectin could adsorb on the surface of positively charged CAS and stabilize the complex through electrostatic forces and steric hindrance [18]. The stability of CAS could be enhanced by adding an appropriate amount of LMP under acidic conditions [19]. The LMP/CAS complex can be obtained by compounding CAS and LMP in a certain proportion. LMP/CAS is a promising food ingredients, which has potential tobe widely used in the food industry.

It is of great significance to study the effects of salt ions on the physical and chemical properties, rheological properties, and microstructure of the typical polysaccharide/protein complex. Some scholars have discussed the influence of a certain salt ion on this complex system. However, to the best of our knowledge, there is no comparative study that has reported the effects of multiple ions on the polysaccharide/protein complex system.

In this study, the effects of different types and concentrations of salt ions (Na^+^, K^+^, and Ca^2+^) on the physicochemical and rheological properties and the structural characteristics of LMP/CAS complex were studied by analyzing its zeta potential, particle size, turbidity, rheological properties, X-ray diffraction, infrared spectrum, and microstructure. Our findings will provide support in the development of polysaccharide/protein complex food systems, such as dairy products and edible films.

## 2. Materials and Methods

### 2.1. Materials

LMP (from orange peel, esterification degree between 22.0% and 28.0%, product number P9135, galacturonic acid ≥74.0%, methoxyl group ≥6.7% under dry condition, *w*/*w*) was supplied by Sigma-Aldrich company (St. Louis, MO, USA). CAS (14.1% protein nitrogen, 90% protein, 5.5% water, 3.8% ash, and 0.02% calcium, *w*/*w*) was supplied by Sigma-Aldrich company (St. Louis, MO, USA). Analytical grade sodium chloride, potassium chloride, calcium chloride, and citric acid were supplied by Sigma Aldrich (Shanghai) Trading Co., Ltd. (Shanghai, China). All solutions were prepared in distilled water.

### 2.2. Sample Preparation

First, LMP (1.0 g) and CAS (0.5 g) were dissolved in 100.0 mL distilled water, bathed in hot water at 80.0 °C for 60 min, with continuous stirring to ensure that the solute was fully dissolved. The solutions were cooled to room temperature (approximately 25.0 °C). Then, the pH was adjusted to 3.0 with citric acid to obtain the LMP/CAS complex. Na^+^, K^+^, and Ca^2+^ with amount-of-substance concentrations of 5, 15, and 30 mM were added to the LMP/CAS complex respectively. Therefore we obtained the Na^+^-LMP/CAS complexes with the concentration of 5, 15, and 30 mM, the same for K^+^-LMP/CAS and Ca^2+^-LMP/CAS. All samples were stored in a refrigerator at 4.0 °C for 24 h for the following analysis. Before each measurement, it was essential to increase the temperature of the samples to room temperature (approximately 25.0 °C) to avoid the influence of temperature on the results. Therefore samples were left at room temperature (approximately 25.0 °C) for a minimum of 60 min [20].

### 2.3. Physical and Chemical Properties

#### 2.3.1. Zeta Potential and Particle Size

The method described by Jia, You, Hu, Liu et al. [21] was used. The Malvern nanoparticle size analyzer (Nano-ZS & MPT-2, Malvern, UK) was used to determine the zeta potential and particle size of samples at 25 °C. The refractive index of solute was 1.543. The absorption rate was 0.001. The sample was diluted to 1000 times. Diluent (1mL) was injected it into the measuring container. After standing still for 2 min, the particle size and zeta potential of LMP/CAS complex samples were measure. The measurements were repeated 6 times for each sample.

#### 2.3.2. Turbidity

The method reported by Wang, Souihi et al. [5] was used with some modifications. Briefly, a turbidity meter (2100AN, HACH Inc., Loveland, CO, USA) was used to measure the turbidity of all samples. After preheating for 30 min, the instrument was calibrated with a standard solution at 600 nm, and 50.0 mL of the solution was injected into the sample cup. The sample cup was placed in the colorimetric cell, and the turbidity value was determined once a stable reading was acquired.

### 2.4. Rheological Properties

#### 2.4.1. Steady Shear Flow Characteristics

Rheological measurements of the LMP/CAS complex were performed using a rotary rheometer (AR-G2, TA instruments Inc., New Castle, DE, USA) following the method described by Agoda Tandjawa, Durand et al. [22] with some modifications. The steady shear flow of the sample was measured using a flat plate measuring system, and the gap was set at 0.5 mm. The diameter of the parallel plate used in the analysis was 60 mm. The equilibrium time of each sample was selected as 180 s, and the temperature was chosen set at 25.0 °C. The shear rate was increased gradually from 0–300 s^−1^ and then decreased gradually from 300–0 s^−1^. The change of shear stress with the shear rate was recorded and the hysteresis loop area (△Hr) was obtained.

#### 2.4.2. Frequency Sweep

A frequency sweep test was used to determine the storage modulus (G′) and loss modulus (G′′) of samples under a small amplitude oscillatory shear [23]. Before the test, the linear viscoelastic region of the solution was determined using a strain sweep (0.01–100%) test with a constant frequency of 1 Hz. Then, the oscillation frequency was set from 0.1–10 Hz, and changes in G′, G′′, and the loss tangent (tanδ) of different samples under 0.5% strain amplitude were determined.

### 2.5. X-ray Diffraction (XRD)

Different LMP/CAS complex solutions (100.0 mL) were collected and spread in Petri dishes. After pre-freezing in the refrigerator for 24 h, the samples were freeze-dried (CASientz-10ND, Ningbo Academy of Sciences, China) at −40 °C for 48 h. Next, XRD was measured using the X-ray diffractometer (X’Pert3 Powder, PANalytical B.V., Almelo, Netherlands) following a previously reported method [24].

### 2.6. Fourier-Transform Infrared Spectroscopy (FT-IR)

The infrared spectra were acquired using a FT-IR spectrometer (Spectrun100, PerkinElmer Inc., Waltham, MA, USA) following the method adapted from Choi and Han [25]. The sample powder (2 mg) was mixed with KBr powder (400 mg) and compressed into a 1-mm-thick disk for scanning. The spectra were obtained in the range of 4000–600 cm^−1^. The scanning frequency was 32 times, and the resolution was 4 cm^−1^.

### 2.7. Scanning Electron Microscopy (SEM)

The scanning electron microscope (SU3500010102, HITACHI, Ltd., Tokyo, Japan) was used to examine the surface morphology and fracture surface of the complex samples following the method reported by Bakhshabadi et al. [26] with some modifications. Briefly, the dried samples were coated with gold using a vacuum sputtering gold-plating machine. The microstructure of samples was observed using SEM at an accelerating voltage of 10 kV under 500× magnification. 

### 2.8. Statistical Analysis

All experiments were conducted in triplicate and data were reported as “mean ± standard deviation”. The analysis of data was performed using one-way analysis of variance (ANOVA) in SPSS 22.0 software (SPSS Korea, Data Solution, Seoul, Korea). Duncan’s Multiple Range Test (DMRT) was used to determine the least significant difference of means, and *p* < 0.05 was statistically significant.

## 3. Results and Discussion

### 3.1. Physical and Chemical Properties of the LMP/CAS Complex

**Figure 1:**
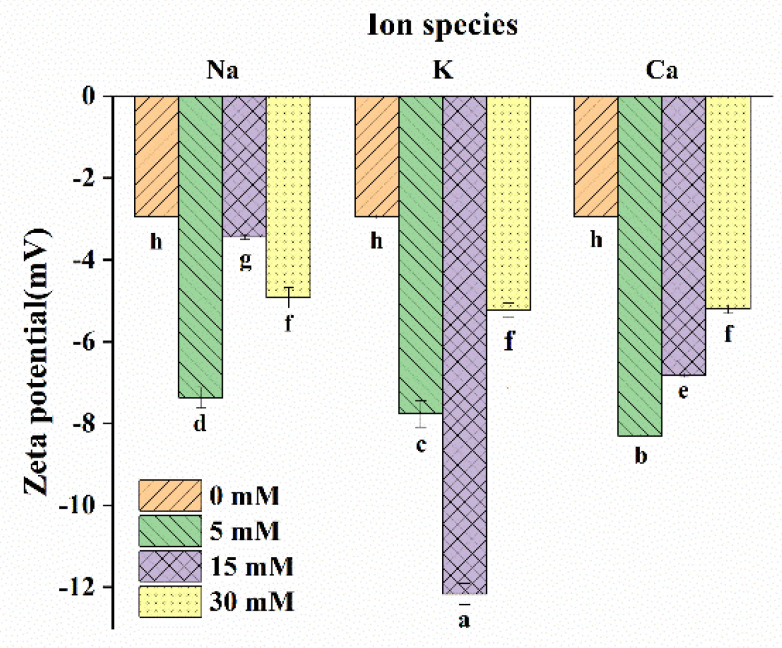
Effect of salt ion species and concentration on the potential of the low methoxyl pectin/sodium caseinate (LMP/CAS) complex. Different letters below the bars indicate a significant difference (*p* < 0.05).

Zeta potential is closely related to the stability of the LMP/CAS complex. As shown in Figure 1, all samples were negatively charged, and the absolute value of zeta potential after the addition of the salts was significantly higher, indicating that the presence of the three salt ions improved the stability of the LMP/CAS complex, but the improvement effect achieved using different salt ions was different. When the concentration of salt ions was 5 mM, there was no significant difference among Na^+^, K^+^, and Ca^2+^ on the zeta potential. However, at a concentration of 15 mM, the zeta potential of Na^+^-LMP/CAS was the lowest and that of K^+^-LMP/CAS was the highest, indicating that K^+^ could improve the stability of the complex at the appropriate concentration. After the addition of Na^+^, the zeta potential first decreased over the concentration range of 5 mM to 15 mM, and then increased over the concentration range of 15 mM to 30 mM. On the other hand, after the addition of K^+^, the zeta potential first increased and then decreased. In the range of 5–30 mM, zeta potential decreased gradually with the addition of Ca^2+^. These findings can be explained on the basis that both Na^+^ and K^+^ could compete with the positively charged reactive groups of CAS and adsorb on the LMP side chain [1] with the formation of a more stable complex. With an increase in Na^+^ concentration, the electrostatic shielding effect increased gradually, and the solubility of CAS also increased gradually [27]. When the concentration was 15 mM, the influence of the latter was more obvious. However, when the concentration continued to increase, the electrostatic shielding effect increased significantly, resulting in the increases in the hydrophobic interaction between LMP and CAS. As for K^+^, the electrostatic shielding effect between K^+^ and CAS also increased with the increase in the concentration. When the concentration reached 30 mM, K^+^ may bind to the carboxyl end of CAS, which lead to the decrease of the binding between CAS and LMP, thus reducing the intermolecular interaction. Differently, Ca^2+^ not only crosslinks with LMP in the complex but also forms a Ca^2+^ bridge with CAS [12]. The role of the latter is more obvious with an increase in Ca^2+^ concentration, which led to the crosslinking degree of Ca^2+^-LMP/CAS complexes decreased, and thereby, the zeta potential of this complex decreased gradually.

**Figure 2:**
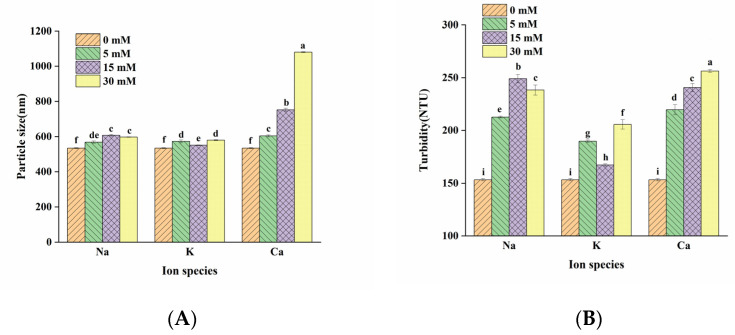
Effect of salt ion species and concentration on particle size (**A**) and turbidity (**B**) of the low methoxyl pectin/sodium caseinate (LMP/CAS) complex. Different letters above the bars indicate a significant difference (*p* < 0.05).

Changes in particle size can reflect the corresponding change in properties of the LMP/CAS complex. As shown in Figure 2A, the particle size of the LMP/CAS complex samples after the addition of the salts was significantly higher than the complex without salt addition, which was probably due to the adsorption of salt ions on the surface of the complex, increasing in particle size. At the same ion concentration, the particle size of Na^+^-LMP/CAS was significantly higher than that of K^+^-LMP/CAS, and the particle size of Ca^2+^-LMP/CAS was significantly higher than that of Na^+^-LMP/CAS and K^+^-LMP/CAS. These findings might be explained on that both Na^+^ and K^+^ played the role of electrostatic shielding. At the same ion concentration, the electrostatic shielding effect of Na^+^ was not as good as that of K^+^ ion, which led to the relative enhancement of electrostatic repulsion of Na^+^-LMP/CAS complexes [1], and thus made larger particle size than K^+^-LMP/CAS complexes. It has been reported that Ca^2+^ can promote the unfolding and aggregation of protein by enhancing hydrophobic interactions and forming a Ca^2+^ bridge, thus prominently enhancing the particle size of the polysaccharide/protein complex [8,23]. Therefore, the particle size of Ca^2+^-LMP/CAS complexes was significantly larger than the Na^+^-LMP/CAS and K^+^-LMP/CAS complexes.

The effects of Na^+^, K^+^, and Ca^2+^ on the turbidity of the LMP/CAS complex are shown in Figure 2B. It can be seen that the turbidity of Na^+^-LMP/CAS, K^+^-LMP/CAS, and Ca^2+^-LMP/CAS complexes were significantly higher than the LMP/CAS complexes. The change in turbidity was found to be consistent with the change in particle size. Studies have shown that the larger the particle size of the complex, the lower the light transmittance [21]. With an increase in ion concentration, the influence of Na^+^ and Ca^2+^ on the turbidity of the complex was significantly greater than that of K^+^, which was explained as the change in particle size. At the same concentration, the turbidity value of the Ca^2+^–LMP/CAS complex was significantly higher than that of the Na^+^–LMP/CAS and K^+^–LMP/CAS complexes. This may be due to the chelation between LMP and Ca^2+^, which led to a more significant increase in the suspended particles in the system [28].

### 3.2. Rheological Properties of the LMP/CAS Complex

#### 3.2.1. Steady Shear Flow Characteristics

**Figure 3:**
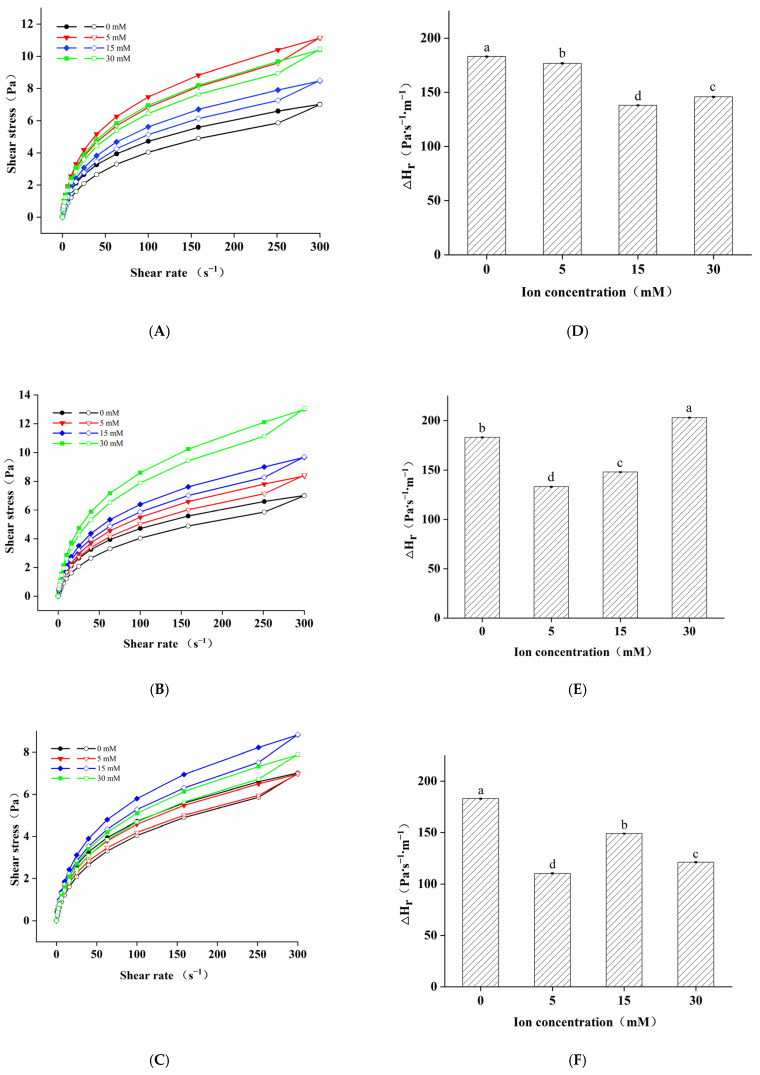
Steady-shear flow properties of the low methoxyl pectin/sodium caseinate (LMP/CAS) complex. Shear stress and hysteresis loop area of Na^+^–LMP/CAS (**A**,**D**), K^+^–LMP/CAS (**B**,**E**), and Ca^2+^–LMP/CAS (**C**,**F**). Different letters above the bars indicate a significant difference (*p* < 0.05).

The steady shear flow characteristics of the LMP/CAS complex are shown in Figure 3A–C. All LMP/CAS complexes were shear-thinning fluids. Moreover, the shear stress of the complex with the added salt ions was higher than that of the control group, which might be related to the improved stability of the complex with addition of salts. We found that with an increase in Na^+^ concentration, the shear stress of Na^+^-LMP/CAS decreased from 5 mM to 15 mM but increased from 15 mM to 30 mM (Figure 1A). It is possible that when the concentration of Na^+^ was 15 mM, the effect of Na^+^ on increasing the solubility of CAS was more obvious than that of electrostatic shielding, thus reducing the binding probability between the protein and polysaccharide molecules and altering the viscosity of the complex to a certain extent [29]. On the contrary, when the concentration increased to 30 mM, the effect of electrostatic shielding was more obvious, so the shear stress increased again. With an increase in K^+^ concentration, the shear stress of K^+^-LMP/CAS was found to increase (Figure 1B). Previous studies have confirmed that K^+^ can effectively shield the repulsion between the carboxyl side chains of LMP molecules and promote the formation of the internal network structure of the complex [30]. The shear stress of the complex increased initially and then decreased with the increase in the concentration of added Ca^2+^ (Figure 1C). At low Ca^2+^ concentration, the complex exhibited rheological properties similar to that of CAS. With an increase in concentration, some of the carboxyl groups of LMP formed a Ca^2+^ bridge via interaction with the positively charged groups of CAS, and the complex had rheological properties similar to that of LMP [31].

The thixotropic loop and the change in the loop area of the LMP/CAS complex are shown in Figure 3D–F. The area of the thixotropic loop reflects the extent of thixotropy. The shear stress of all samples depicts thixotropic loops, indicating that they are all thixotropic systems. Except for K^+^-LMP/CAS with ion concentration of 30 mM, the areas of the thixotropic loops of other samples with added salt ions were significantly lower than those of the control group. The thixotropic loop area of the Na^+^-LMP/CAS complex followed the order of Na^+^ concentration of 5 mM > 30 mM > 15 mM. The thixotropic loop area of K^+^-LMP/CAS increased as the concentration was increased from 5 mM to 30 mM, whereas that of Ca^2+^-LMP/CAS increased initially and then decreased, which was consistent with the stress change after the addition of salt ions. At a K^+^ concentration of 30 mM, the area of the thixotropic ring was higher than that of the control group, which indicated that the high concentration of K^+^ could increase the thixotropy of the LMP/CAS complex. It is likely that because a high K^+^ concentration have a special effect with CAS, which changed the structure of the K+-LMP/CAS complex [30].

#### 3.2.2. Frequency Sweep Measurements

**Figure 4:**
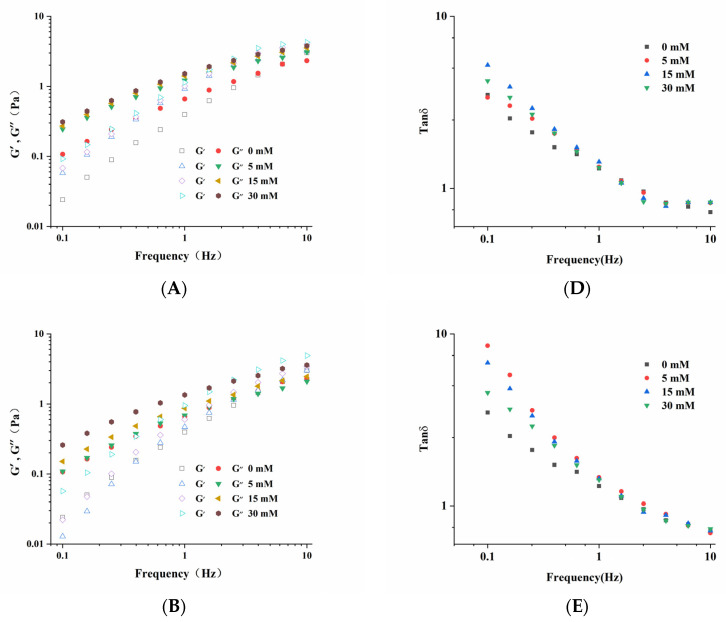

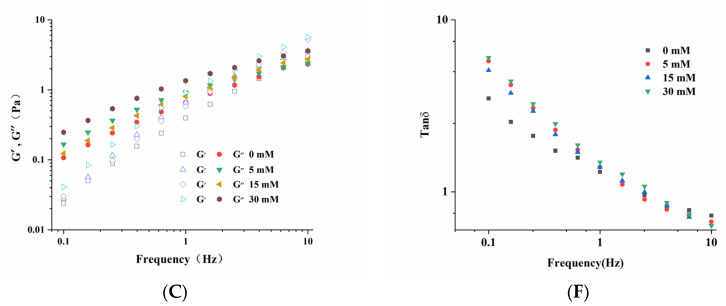
Viscoelastic behavior of the low methoxyl pectin/sodium caseinate (LMP/CAS) complex. Storage modulus (G’) and loss modulus (G”) of Na^+^–LMP/CAS (**A**), K^+^–LMP/CAS (**B**), and Ca^2+^–LMP/CAS (**C**). Loss tangent of Na^+^–LMP/CAS (**D**), K^+^–LMP/CAS (**E**), and Ca^2+^–LMP/CAS (**F**).

Figure 4 shows the viscoelastic behavior of the LMP/CAS complex. G′ is the storage modulus, representing the energy stored in the complex system and reflecting the elasticity of the system. G′′ represents the energy lost due to irreversible viscous deformation, reflecting the viscosity of the system. Tanδ is the loss tangent value, which is the ratio of G′′ to G′. As shown in Figure 4A–C, G′ and G′′ had a higher frequency dependence at low angular frequency but less dependence at a higher frequency. After the addition of salt ions, the G′ and G′′ of samples were higher, indicating that the structure of the LMP/CAS complex was strengthened after the addition of Na^+^, K^+^, and Ca^2+^. At the same time, as the concentrations of Na^+^, K^+^, and Ca^2+^ were increased from 5 mM to 30 mM, the G′ and G′′ increased, and the results were consistent with those from previous studies [31]. Figure 4D–F shows the changes in tanδ of the LMP/CAS complex containing Na^+^, K^+^, and Ca^2+^, respectively. The tanδ of samples containing Na^+^, K^+^, and Ca^2+^ was significantly higher than that of the control group, indicating that the addition of these three salt ions could improve the fluidity of the LMP/CAS complex. This result can be explained on the basis that the molecular chain movement of the complex may be easier owing to the addition of salt ions [23,32]. It can be seen that tanδ is more than 1 in most cases over the measurement range, indicating that the complex exhibits liquid characteristics. When the concentration of Na^+^ was increased from 5 mM to 15 mM, the tanδ of the complex increased, while when the concentration of Na^+^ was increased from 15 mM to 30 mM, the tanδ decreased, indicating that a specific concentration of Na^+^ could strengthen the structure of the complex [27]. With an increase in K^+^ concentration, the tanδ of the complex decreased, indicating that the proportion of viscous components in the complex decreased with an enhancement of stability [1]. The decrease of the tanδ of the Na^+^-LMP/CAS and K^+^-LMP/CAS complexes may be attributed to the increase in elasticity caused by the strengthening of macromolecular network structure [33]. However, when the Ca^2+^ concentration was increased, tanδ decreased initially and then increased compared with that observed at low Ca^2+^ concentrations. The excess Ca^2+^ may form a calcium bridge with CAS and affect the structure of the LMP-CAS complex to a certain extent, thereby increasing the fluidity of the complex.

### 3.3. X-ray Diffraction (XRD)

**Figure 5:**
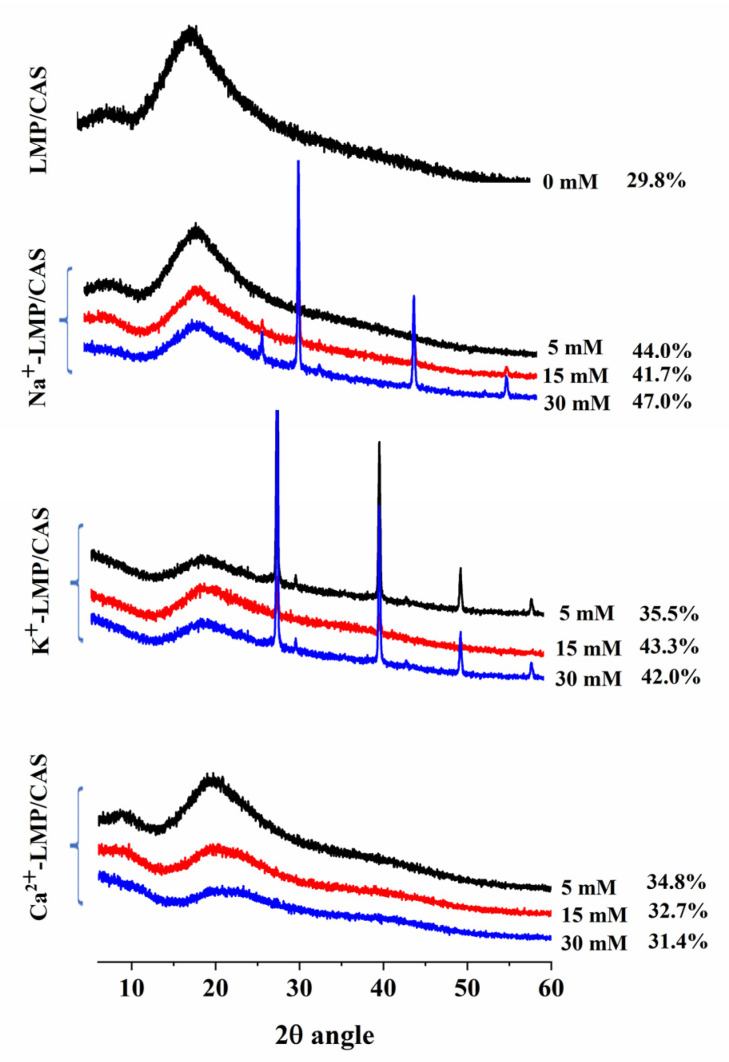
The X-ray diffractograms and the crystallinity of the low methoxyl pectin/sodium caseinate (LMP/CAS) complex.

Diffraction peak intensity can reflect the grain size of the crystalline region of the sample [23]. The XRD pattern of the LMP/CAS complex is shown in Figure 5. The diffraction peak of the LMP/CAS complex was observed at about 20°, and the LMP/CAS complex was approximately amorphous. The diffraction peak intensity of LMP/CAS complexes was significantly higher after the addition of Na^+^, K^+^, and Ca^2+^. Some studies have reported that salt ions can change the diffraction peak intensity of proteins during heating [34]. We found that when these three ions were used at the same concentration, the diffraction peak intensity of Na^+^-LMP/CAS was the highest and that of Ca^2+^-LMP/CAS was the lowest. Moreover, the ion diffraction peaks were clearly visible in the XRD patterns of Na^+^-LMP/CAS and K^+^-LMP/CAS, but not observed in Ca^2+^-LMP/CAS. This finding indicated that the complex gradually changed to a crystalline structure after the addition of Na^+^ and K^+^, while the complex was still amorphous after adding Ca^2+^.

The crystallinity of samples was calculated based on the XRD findings, and the results are listed in Figure 5. Through the calculation of the crystallinity, we can more clearly see the changes in the crystal structure of LMP/CAS complexes with the three ions at different concentrations. The crystallinity of the LMP/CAS complex was 29.8% and increased significantly after the addition of the salt ions, but different concentrations and different types of ions lead to varying increases. The crystallinity of Na^+^-LMP/CAS increased by 11.9–17.2% compared with that of the LMP/CAS complex; the crystallinity of K^+^-LMP/CAS increased by 5.7–13.5% and that of Ca^2+^-LMP/CAS increased by 1.6–5.0%. The variation of crystallinity with increases in salt concentration was consistent with that of the zeta potential. The reason for this phenomenon may be that the structure of the complex was more orderly after the addition of salt ions [24]. Moreover, the effect of salt ions on crystallinity followed the order of Na^+^ > K^+^ > Ca^2+^.

### 3.4. Fourier Transform-Infrared (FT-IR) Spectroscopy

**Figure 6:**
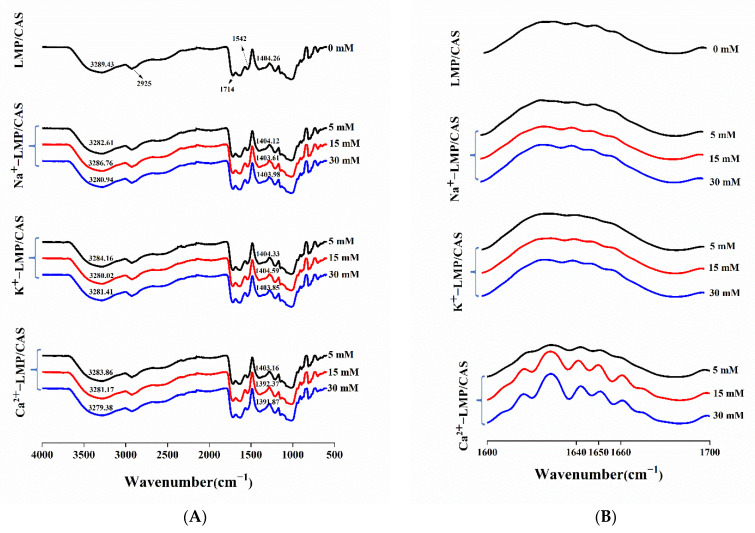
FT-IR spectra (**A**) and spectra of amide I band region (**B**) of the low methoxyl pectin/sodium caseinate (LMP/CAS) complex.

The effects of different concentrations of Na^+^, K^+^, and Ca^2+^ on the chemical structure of the LMP/CAS complex were studied using FT-IR (Figure 6A). It can be seen that the infrared peaks of all samples are almost the same. There is no new peak in the FT-IR spectrum of the LMP/CAS complex after the addition of the salt ions, indicating that the three salts do not induce the formation of a new covalent bond in the complex when in solution. The peaks near 3289 cm^−1^ and 2925 cm^−1^ correspond to the hydrophilic O-H and hydrophobic C-H, respectively, which represent the characteristic peaks of the LMP and CAS combination [25]. The peaks from 2000 cm^−1^ to 2500 cm^−1^ mainly represent the stretching vibration of the triple bonds such as C≡C and C≡N and the accumulated double bonds, and the absorption peak near 1723 cm^−1^ represents the stretching of the esterified carbonyl group (C=O). Due to the intermolecular interactions between the carboxylic acid groups in LMP and amide groups in CAS, an absorption peak is observed near 1543 cm^−1^ [35]; the absorption peak near 1404 cm^−1^ is attributed to the N-H bending vibration, and multiple weak vibrations between 950 cm^−1^ and 1300 cm^−1^ correspond to the characteristic peaks of pectin [36].

The characteristic O-H peaks of all samples containing Na^+^, K^+^, and Ca^2+^ salt ions showed a blue shift and enhanced absorption peak intensity compared with those of the control group, indicating that the addition of salt ions affected the electrostatic interaction between pectin and protein [24], resulting in the formation of more hydrogen bonds in the complex, strengthening the intermolecular force, and making the LMP/CAS complex more stable. However, the blue shift resulting from the addition of different types of salt ions at different concentrations varied. With an increase in the salt ion concentration from 5 mM to 30 mM, the degree of blue shift in Na^+^-LMP/CAS decreased initially and then increased, whereas that in K^+^-LMP/CAS increased initially and then decreased, which was consistent with the results from the macroscopic analysis. However, the degree of blue shift in Ca^2+^-LMP/CAS increased with an increase in the salt ion concentration and may be related to the crosslinking of Ca^2+^ and LMP [12]. Previous studies have shown that the addition of a small amount of salt ions can shield intramolecular repulsion, promote short-range intermolecular electrostatic attraction, and help form the condensed complex [21,37]. The absorption peak of N-H in Ca^2+^-LMP/CAS showed a significant blue shift when the concentration of Ca^2+^ was 15 mM and 30 mM, which was about 12 cm^−1^ at 15 mM and 13 cm^−1^ at 30 mM, and likely related to the Ca^2+^ bridge formed between Ca^2+^ and CAS, and the hydrogen bond in the complex [38]. Therefore, it can be speculated that Ca^2+^ affects the structure of proteins in the complex.

Figure 6B shows the absorption spectrum of the LMP/CAS complex in the amide I band region, and Table 1 lists the relative contents of four different secondary structures of proteins in the complex. Among them, the α-helix is represented at 1650~1658 cm^−1^, β-fold at 1600~1640 cm^−1^, β-turn at 1660~1700 cm^−1^, and random coil at 1640~1650 cm^−1^ [39]. The α-helix conformation and β-fold represent an ordered structure and the β-turn is the secondary structure connecting them. The random coil represents a disordered structure. It can be seen that the contents of the four secondary structures of the samples containing different concentrations of Na^+^ and K^+^ are not significantly different from those of the control group, indicating that the addition of these two ions does not change the protein conformation in the LMP/CAS complex. With addition of Ca^2+^, the α-helix content decreased, and the β-fold and β-turn contents increased, while the content of the random coil did not change significantly. These findings indicated that the spatial structure of CAS was partially unfolded, some α-helices were disintegrated after the interaction between Ca^2+^ and CAS, and the β-folds and β-turns formed after rearrangement. With addition of Ca^2+^ at a low concentration (5 mM), α-helix mainly shifted towards β-turn. At a medium to high level of Ca^2+^ addition (15 and 30 mM), content of β-fold increased. This result suggested that Ca^2+^ affected the secondary structure of CAS in the LMP/CAS complex.

### 3.5. Microstructure Analysis

**Figure 7:**
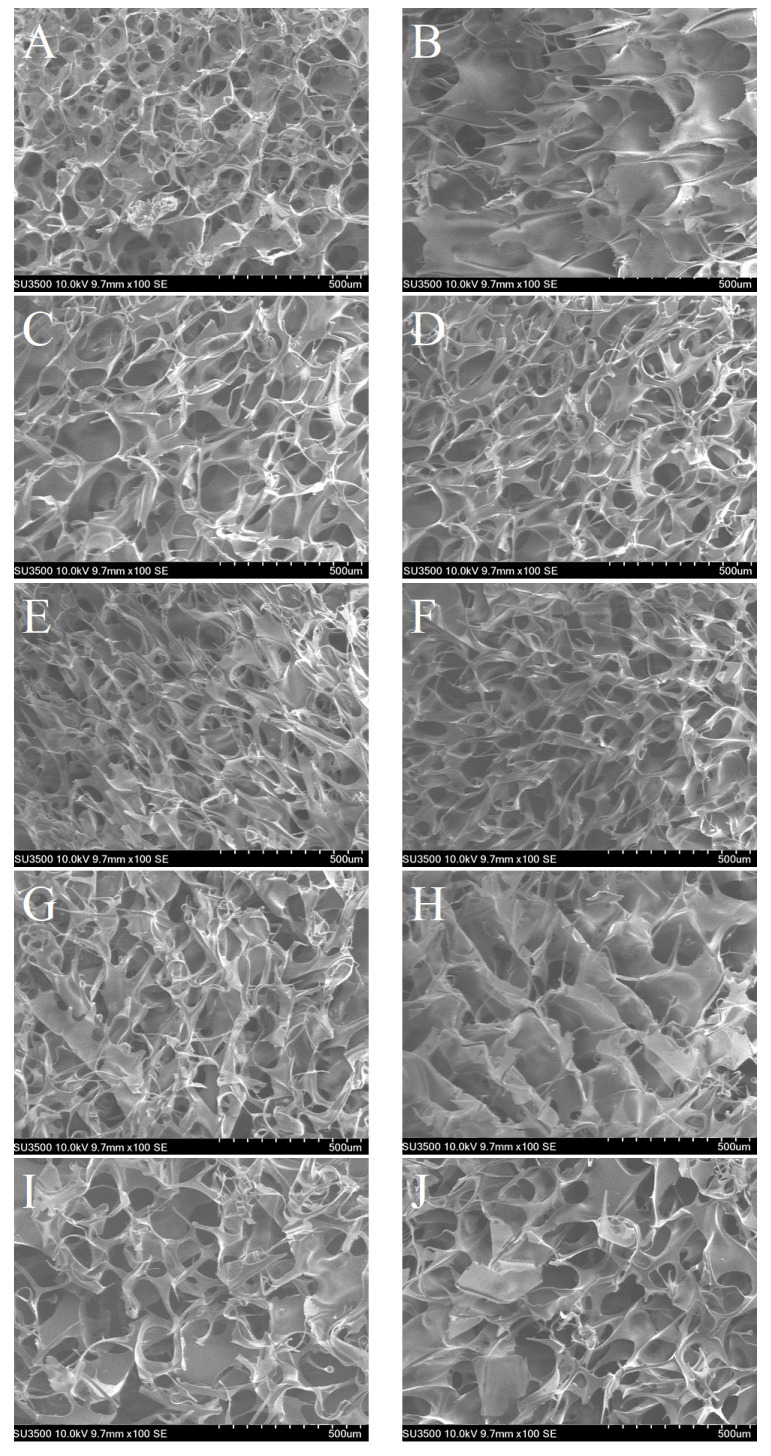
SEM images of the low methoxyl pectin/sodium caseinate (LMP/CAS) complex. (**A**) represent LMP/CAS complexes. (**B**–**D**) represent LMP/CAS complexes with the addition of 5, 15, and 30 mM Na^+^, respectively. (**E**–**G**) represent LMP/CAS complexes with the addition of 5, 15, and 30 mM K^+^, respectively. (**H**–**J**) represent LMP/CAS complexes with the addition of 5, 15, and 30 mM Ca^2+^, respectively.

The microstructure of the LMP/CAS complex containing different concentrations of Na^+^, K^+^, and Ca^2+^ was visualized using SEM. As shown in Figure 7A, the LMP/CAS complex was found to have an irregular network structure. When salt ions were added, the three-dimensional structure of the complex tended to be orderly (Figure 7B–J). However, different types and concentrations of salt ions exhibited different effects, which are discussed subsequently.

As shown in Figure 7B, Na^+^-LMP/CAS has a clear layered structure when the concentration of the added salts was 5 mM. When the salt ion concentration was 15 mM (Figure 7C) and 30 mM (Figure 7D), the complex exhibited a change from a layered to a denser and more uniform network structure, and the antenna-shaped structure could be clearly seen on the surface of Na^+^-LMP/CAS complexes, which may be due to the addition of Na^+^, the tensile and expansion effect of the complex on CAS was weakened, and the curling effect was enhanced, which led to the decrease of the size of network structure and the increase in the number of entangled structures [29]. Similar results of the complex exhibiting a dense entangled structure were reported for the cellulose/LMP complex after the addition of NaCl [22].

When 5 mM K^+^ was added, the layered structure on the surface of the complex was visible (Figure 7E), and when the concentration was increased to 15 mM, a compact and regular network structure of the K^+^-LMP/CAS complex was observed (Figure 7F). Our findings are consistent with the results published in the literature and suggest that appropriate K^+^ concentrations can lead to the formation of a dense porous network structure by promoting hydrogen bonding [40]. However, when the concentration was increased to 30 mM, an irregular coiled and folded structure appeared in the network structure on the surface of the K^+^-LMP/CAS complex (Figure 7G), likely because the excess K^+^ had a special effect with CAS, thereby reducing the hydrogen bonding and hydrophobic interaction between the pectin chain and CAS [27].

When the concentration of Ca^2+^ was 5 mM, a uniform and loose network formed within the LMP/CAS complex (Figure 7H). Previous studies have shown that the right amount of salt ions can make the LMP/CAS complex have a more regular and uniform network structure and a tighter cavity, and improve the stability of the complex [41]. When the Ca^2+^ concentration increased from 15 mM to 30 mM, it was observed that several massive structures formed in the complex and became more obvious at a higher concentration (Figure 7I,J). This phenomenon may likely be related to the calcium bridge formed between Ca^2+^ and CAS, thereby explaining the change in tanδ as shown in Figure 4.

## 4. Conclusions

In this study, we found that the appropriate concentrations of Na^+^, K^+^, and Ca^2+^ could result in a more regular and uniform network structure formed by the LMP/CAS complex and improve the stability and rheological properties of the complex. Among the salt ions, Na^+^ and K^+^ affected the LMP/CAS complex through electrostatic adsorption with LMP and electrostatic shielding of CAS, while Ca^2+^ through crosslinking with LMP and forming Ca^2+^ bridge with CAS. This study provides a reference for the use of the LMP/CAS complex in the food industry. For practical applications, the appropriate type and concentration of salt ions should be selected based on the product type and formulation. For example, low concentration Ca^2+^ could be added when making LMP/CAS complex dairy products with high stability and good fluidity. High concentration K^+^ could be added when making LMP/CAS complex edible films with high material rigidity requirements. Future studies can focus on the use of these types and concentrations of ions for specific applications of foods composed of the LMP/CAS complex and the mechanism of the effect of salt ions on the complex system.

## Figures and Tables

**Table 1:** The relative contents of four different secondary structures of protein in the low methoxyl pectin/sodium caseinate (LMP/CAS) complex.

Samples	Ion Concentration (mM)	α-Helix (%)	β-Fold (%)	β-Turn (%)	Random Coil (%)
LMP/CAS	0	26.14 ± 0.31 ^a^	48.07 ± 0.14 ^b^	10.09 ± 0.34 ^cd^	15.70 ± 0.17 ^a^
Na^+^-LMP/CAS	5	26.63 ± 0.59 ^a^	47.87 ± 1.29 ^bc^	9.81 ± 0.20 ^cd^	15.69 ± 0.51 ^a^
	15	26.32 ± 0.32 ^a^	47.77 ± 0.72 ^bc^	9.75 ± 0.07 ^d^	16.16 ± 0.33 ^a^
	30	26.65 ± 0.04 ^a^	46.70 ± 0.01 ^bc^	10.07 ± 0.09 ^cd^	16.57 ± 0.05 ^a^
K^+^-LMP/CAS	5	26.39 ± 0.16 ^a^	47.23 ± 0.41 ^bc^	9.95 ± 0.60 ^cd^	16.43 ± 0.35 ^a^
	15	26.97 ± 0.06 ^a^	46.22 ± 0.23 ^c^	10.63 ± 0.19 ^c^	16.19 ± 0.35 ^a^
	30	26.57 ± 0.12 ^a^	47.02 ± 0.13 ^bc^	10.68 ± 0.05 ^c^	15.74 ± 0.04 ^a^
Ca^2+^-LMP/CAS	5	14.71 ± 0.05 ^b^	48.30 ± 0.18 ^b^	20.67 ± 0.10 ^a^	16.33 ± 0.24 ^a^
	15	13.93 ± 0.24 ^bc^	50. 48 ± 0.26 ^a^	19.25 ± 0.14 ^b^	16.09 ± 0.61 ^a^
	30	13.60 ± 0.09 ^c^	51.50 ± 0.14 ^a^	18.97 ± 0.19 ^b^	15.93 ± 0.04 ^a^

All results were expressed as mean ± SD, *n* = 3. Different lowercase letters in the same column indicate a significant difference (*p* < 0.05).

## Data Availability

Not applicable.
